# Plant Community Traits Respond to Grazing Exclusion Duration in Alpine Meadow and Alpine Steppe on the Tibetan Plateau

**DOI:** 10.3389/fpls.2022.863246

**Published:** 2022-07-04

**Authors:** Tianyu Zhan, Wenwu Zhao, Siyuan Feng, Ting Hua

**Affiliations:** ^1^State Key Laboratory of Earth Surface Processes and Resource Ecology, Faculty of Geographical Science, Beijing Normal University, Beijing, China; ^2^Faculty of Geographical Science, Institute of Land Surface System and Sustainable Development, Beijing Normal University, Beijing, China

**Keywords:** grazing exclusion, vegetation restoration, alpine meadow, alpine steppe, the Tibetan Plateau

## Abstract

Grazing exclusion has been a primary ecological restoration practice since the implement of “Returning Grazing Land to Grassland” program in China. However, the debates on the effectiveness of grazing exclusion have kept for decades. To date, there has been still a poor understand of vegetation restoration with grazing exclusion duration in alpine meadows and alpine steppes, limiting the sustainable management of grasslands on the Tibetan Plateau. We collected data from previous studies and field surveys and conducted a meta-analysis to explore vegetation restoration with grazing exclusion durations in alpine meadows and alpine steppes. Our results showed that aboveground biomass significantly increased with short-term grazing exclusion (1–4 years) in alpine meadows, while medium-term grazing exclusion (5–8 years) in alpine steppes (*P* < 0.05). By contrast, belowground biomass significantly increased with medium-term grazing exclusion in alpine meadows, while short-term grazing exclusion in alpine steppes (*P* < 0.05). Long-term grazing exclusion significantly increased belowground biomass in both alpine meadows and alpine steppes. medium-tern, and long-term grazing exclusion (> 8 years) significantly increased species richness in alpine meadows (*P* < 0.05). Only long-term GE significantly increased Shannon-Wiener index in plant communities of alpine steppes. The efficiency of vegetation restoration in terms of productivity and diversity gradually decreased with increasing grazing exclusion duration. Precipitation significantly positively affected plant productivity restoration, suggesting that precipitation may be an important factor driving the differential responses of vegetation to grazing exclusion duration in alpine meadows and alpine steppes. Considering the effectiveness and efficiency of grazing exclusion for vegetation restoration, medium-term grazing exclusion are recommended for alpine meadows and alpine steppes.

## Introduction

Grazing exclusion (GE) has become a primary management practice for restoring degraded grasslands since the central government began to implement the program “Returning Grazing Land to Grassland” in 2003 (Lu et al., 2015). However, there have been lots of debates on restoration efficiency of GE during the past nearly two decades after the program “Returning Grazing Land to Grassland” (Sacha, 2020; Sun et al., 2020a). Numerus studies have proven that GE eliminates consumption from livestock and accelerates plant growth so as to significantly improve plant aboveground biomass (Gao et al., 2011; Sun et al., 2018); while plant belowground biomass may not change after GE (Reeder et al., 2004; Niu et al., 2011); or even GE may reduce grassland primary productivity (Cheng et al., 2011). As well, GE could be beneficial for plant species diversity restoration (Bakker et al., 2006; Zhu et al., 2016), or shows little influence in some cases (Lunt et al., 2010; Guo et al., 2019), or even undermine biodiversity targets (Zhao et al., 2019; Sun et al., 2021a). In fact, these inconsistent results about the effects of GE on vegetation restoration depend on the grasslands’ nature and specific situations, for example, grassland type (Cao et al., 2019) and GE duration (Zhang et al., 2021).

GE duration, an important factor driving grassland restoration, has drawn extensive attentions from scholars and policy makers, because grasslands could respond differently to GE with duration even in a particular site (Zhang et al., 2021; Liu et al., 2022). Generally, there would be a rapid restoration of vegetation in the first few years after GE (Li et al., 2017; Zhang et al., 2021; Liu et al., 2022). The restoration efficiency (i.e., restoration effect per unit of time) decreases as GE duration increases, that is, long-term GE has little sustainable benefit or even wrecks grassland renewal (Sun et al., 2021a). The restoration efficiency (Liu et al., 2020a) decreases as GE duration increases, that is, long-term GE has little sustainable benefit or even wrecks grassland renewal (Sun et al., 2021a). Previous studies have proven that long-term GE reduced not only grassland productivity (Jing et al., 2013) but also plant diversity (Xiong et al., 2014; Nishizawa et al., 2016), which further leads to grassland redegradation (Shang et al., 2008; Su et al., 2015). What’s more, divergent types of grasslands with different climatic conditions exhibit different responses to GE duration. For instance, degraded alpine meadow in humid regions can recover well after short-term GE (Liu et al., 2020b); medium-term GE is suitable for recovery of typical steppe in semi-arid regions (Wu et al., 2014b); whereas long-term GE is necessary to restore degraded typical steppe grassland in arid regions (Jing et al., 2013). However, most studies have been scrutinized on grassland restoration with GE duration for only one grassland type at a specific site, limiting a full understanding of effectiveness of grassland restoration by GE.

The Tibetan Plateau covers an area of 2.5 million km^2^ with 50.9% occupied by alpine grasslands, playing an important role in ecological security and economic development, and livestock husbandry (Sun and Wang, 2016). Alpine meadow and steppe are two primary types of grasslands on the Tibetan Plateau. Due to the unique climate and fragile biomes, these alpine grasslands are highly susceptible to climate change and anthropogenic activities and have undergone serious degradation during recent decades, greatly threatening welfare of herdsmen (Harris, 2010; Zhang et al., 2019). There is an absolute need for restoring these degraded alpine meadow and steppe on the Tibetan Plateau. A key goal of grassland restoration is the reestablishment of vegetation, primarily involving the restoration of plant productivity and biodiversity. The former determines grassland carrying capacity and thus livestock husbandry, while the latter is an important factor driving ecosystem stabilization and functioning (Tilman et al., 2001; Pennekamp et al., 2018). It is therefore of great theoretical value and practical significance to explore the common pattern of vegetation restoration with GE duration in both alpine meadow and steppe, in order to facilitate adaptive strategies based on grassland type for mitigating grassland deterioration.

In this study, our main objective was to evaluate how the direction and rate of vegetation restoration varies with GE duration and grassland type. Although the percentage of mean annual changes in belowground biomass, species richness and Shannon-Wiener index was not significantly related to GE duration in alpine meadows (Figures 3B,C) or alpine steppes (Figures 3F,H), aboveground biomass was significantly negatively related to GE duration in both alpine meadows (*R*^2^ = 0.06; *P* < 0.001; Figures 3A) and alpine steppes (*R*^2^ = 0.20; *P* < 0.001; Figure 3E). More specifically we hypothesized that: (i) the patterns of vegetation restoration with GE duration vary between alpine meadow and steppe; (ii) the restoration effectiveness of GE changes with GE duration; and (iii) precipitation is an important driver of restoration effectiveness of GE duration. To test these hypotheses, we collected data (including above- and belowground biomass, species richness, and Shannon-Wiener index) from both previous studies and field surveys describing vegetation restoration conditions with different GE durations in both alpine meadows and alpine steppes.

## Materials and Methods

### Data Collection

**FIGURE 1:**
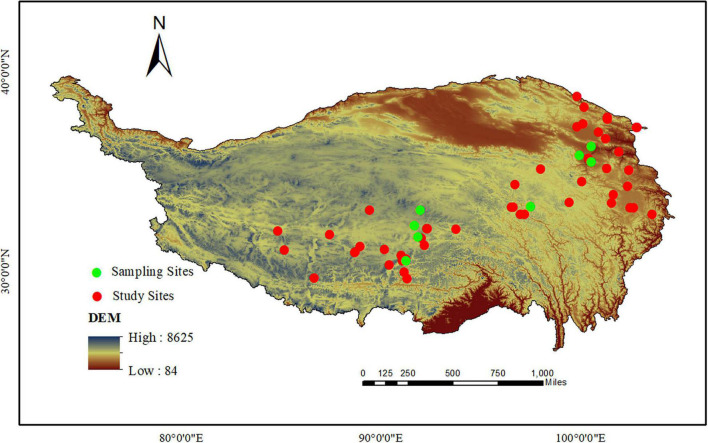
Distribution of sample sites of the data sets included in this study.

We collected peer-reviewed journal articles published before July 2021 from Web of Science and the China Knowledge Resource Integrated Database by using the following search term combinations: “grazing exclusion,” “fence,” “alpine meadow/steppe,” “Qinghai-Tibet/Tibetan Plateau.” Several criteria were set to avoid bias in paper selection: (1) the experimental data should be obtained from field studies with no other treatments (e.g., warming, precipitation change, or nutrient enrichment); (2) GE must be of a duration with at least 1 year; (3) The values without GE is necessarily included as the “reference” to enable the “effect size” analysis; (4) The experimental data in the publications had at least one of the following variables: Plant aboveground biomass (AGB), belowground biomass (BGB), species richness (SR), Shannon-Wiener index (Supplementary Table 1); (5) The mean, standard error or standard deviation, and samples size of selected variables could be acquired directly from tables, digitized graphs, or the body of the text. In total, 43 published papers (Supplementary Table 2) that conformed with these criteria were selected, covering 60 study sites (Figure 1). The GetData Graph Digitizer software (ver.2.25)^1^ was used to extract data from figures in the papers. Additionally, information of study sites on grassland type, longitude, and latitude were also collected. Monthly mean precipitation (MMP) were obtained at 30-s (∼1 km^2^) spatial resolution from 2000 to 2020. These data were provided by National Earth System Science Data Center, National Science and Technology Infrastructure of China.^2^ We extracted the climate data by the Kriging interpolation method using ArcGIS 10.2 (ESRI, Inc., Redlands, CA, United States) for each of sample plots according to their geographic coordinates (latitude and longitude). Mean annual precipitation (MAP) are further calculated by MMP.

Additionally, we selected 9 typical sites in a large-scale transect with different GE duration in both alpine meadows and alpine steppes and conducted the field surveys at the peak growing season in 2020. The information on duration of GE, location, and grassland type in each site were identified and recorded. We divided the duration of GE into short (1–4 years), medium (5–8 years), and long (> 8 years) term according to previous studies (Sun et al., 2020a). Three 50 cm × 50 cm quadrats were randomly selected as replicates for vegetation investigation and sampling inside and outside the fence, respectively. All plant species within each quadrat were identified and their coverage, number, and height were measured and recorded. Then, we clipped the aboveground parts of plants with scissors to determine the aboveground biomass. The belowground parts of plants (soil depth ≤ 30 cm) were obtained from 3 soil cores of 5 cm diameter which were washed to remove residual soil and gain roots with a 0.5 mm sieve. The above- and belowground biomass were determined as constant weights by oven-drying the plant samples at 65°C for 72 h.

### Statistical Analysis

The Shannon-Wiener diversity index (*H*) was calculated as follows (Ma et al., 1995):


$$H=-\sum_{i=1}^{S}\left(P\,i\,\ln P\,i\right)$$

where *S* represents the total number of plant species in the plot, *P*_*i*_ represents the relative importance value of species *i*, calculated as follows (Wang et al., 2014):


$$P_{i}=\left(R\,H+R\,C+R\,B\right)/3$$

where *RH*, *RC*, and *RB* represent height, cover, and biomass of species *i* divided by sum of those for all species, respectively.

We conducted the meta-analysis to determine whether GE with different durations had a significant effect by using the MetaWin 2.1 software (Sinauer Associates Inc., Sunderland, MA, United States) (Hedges et al., 1999; Zhan et al., 2020), in which the response ratio (*RR*) of response variables was calculated to assess the effects of GE as follows:


$$R\,R=\text{Ln}\,\left({\bar{X}}_{t}/{\bar{X}}_{c}\right)$$

where ${\bar{X}}_{t}$ and ${\bar{X}}_{c}$ represent the arithmetic mean concentrations of the concerned variable in GE group (treatment) and that without GE (control), respectively.

The variance (v) of the *RR* was estimated by:


v=St2nt⁢X¯t2+Sc2nc⁢X¯c2

where *n*_*t*_ and *S*_*t*_ represent the sample sizes and the standard deviations of the concerned variable in the treatment group, respectively; *n*_*c*_ and *S*_*c*_ are the sample sizes and the standard deviations of the concerned variable the control group, respectively. The reciprocal of the variance ($w=\frac{1}{v}$) is used as the weight (*W*) for each *RR*. The mean response ratio (*RR*_++_) is calculated from *RR* values of individual pairwise comparisons between the control and treatment group, *RR*_ij_ (*i* = 1,2,3…,*m*; *j* = 1,2,3…,*k*) as follows:


$$R\,R_{++}=\frac{\sum_{i=1}^{m}\sum_{j=1}^{k}w_{i\,j}\,R\,R_{i\,j}}{\sum_{i=1}^{m}\sum_{j=1}^{k}w_{i\,j}}$$

where *m* is the number of treatment groups, and *k* is the number of comparisons in the corresponding control group. The bias-corrected 95% confidence intervals (CIs) were calculated *via* a bootstrapping procedure (5,000 iterations) for each categorical group (Guo and Gifford, 2002). It indicates a statistically significant response of the CIs of the *RR*_++_ did not overlap zero (Zhan et al., 2020). The standard error of *RR*_++_ was estimated by the following equation:


$$\text{S}\,\left(R\,R_{++}\right)=\sqrt{\frac{1}{\sum_{i=1}^{m}\sum_{j=1}^{k}W_{i\,j}}}$$

The vegetation restoration efficiency of GE was illustrated by the percentage of mean annual changes in above- and belowground biomass, species richness, and Shannon-Wiener index (ΔV% yr^–1^) due to GE, which were calculated using the following equations:


$$\Delta \,V\%\,\,y\,r^{-1}=\left(V\,G\,E-V\,F\,G\right)/V\,F\,G^{*}\,100/Y$$

where *V*_*GE*_ and *V*_*FG*_ represent the values of objective variables (above- and belowground biomass, species richness, and Shannon-Wiener index) with and without GE, respectively. *Y* is the duration of GE.

linear-regression analysis were performed to explore the relationships between the percentage of mean annual changes (i.e., vegetation restoration efficiency) in above- and below-ground biomass, and species richness with GE duration and precipitation. The linear-regression analysis were carried out with R version 3.3.2 software (R Core Team, 2016).

## Results

### Changes in Plant Characteristics With the Duration of Grazing Exclusion

**FIGURE 2:**
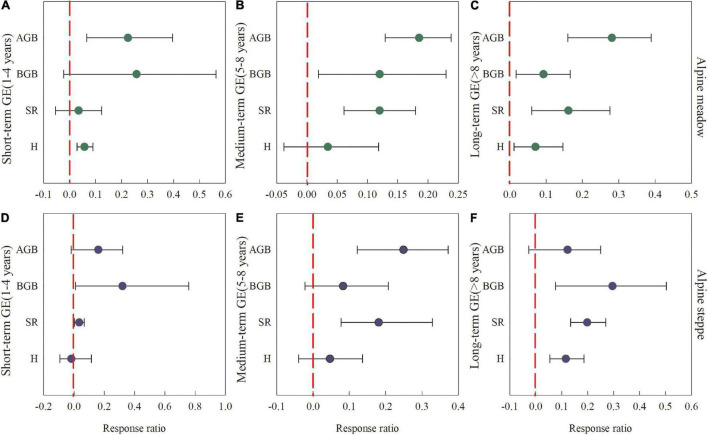
Response ratios (RR) of plant above- and belowground biomass, species richness, and Shannon-Wiener index in response to different grazing exclusion duration in alpine meadows **(A–C)** and alpine steppes **(D–F)**. Grazing exclusion durations were divided into short- [1–4 years; **(A,D)**], medium- [5–8 years; **(B,E)**], and long-term [> 8 years; **(C,F)**], respectively. Error bars are the 95% bootstrapped confidence intervals. AGB, BGB, SR and H represent aboveground biomass, belowground biomass, species richness and Shannon-Wiener index, respectively.

The aboveground biomass significantly increased with short-, medium-, and long-term GE in alpine meadows with the mean response ratios of 0.22, 0.19, and 0.28, respectively (*P* < 0.05, Figures 2A–C), while only increased with medium-term GE in alpine steppes with the mean response ratio of 0.25 (*P* < 0.05, Figures 2D–F). Short-term GE significantly increased belowground biomass with the mean response ratio of 0.32 in alpine steppes (*P* < 0.05, Figure 2D), while showed no significant effect on that in alpine meadows (*P* > 0.05, Figure 2A) where belowground biomass was significantly increased by medium- and long-term GE, with the mean response ratios of 0.12 and 0.09, respectively (*P* < 0.05, Figures 2B,C). Species richness showed a slight (no-significant) increase with short-term GE, while significantly increased by medium- and long-term GE in alpine meadows with the mean response ratios of 0.12 and 0.16, respectively (*P* < 0.05, Figures 2B,C). By contrast, species richness was significantly increased with all durations of GE in alpine steppes, and the mean response ratios were 0.03, 0.18, and 0.20 for short-, medium-, and long-term GE, respectively (*P* < 0.05, Figures 2D–F). Moreover, there were increases in Shannon-Wiener index in alpine meadows with short- and long-term GE and alpine steppes with long-term GE.

### Factors Driving Vegetation Restoration With Grazing Exclusion

**FIGURE 3:**
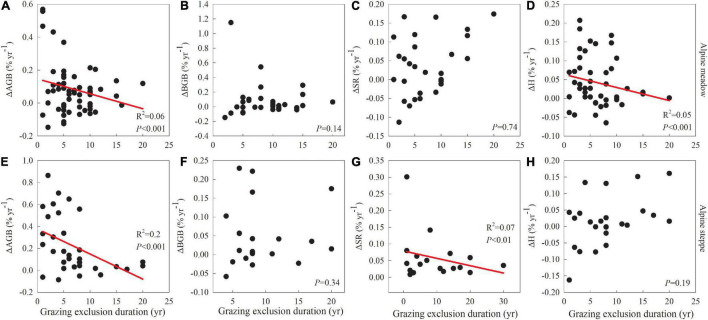
Relationships of the percentage of mean annual changes in above- and belowground biomass, species richness, and Shannon-Wiener index with grazing exclusion duration in alpine meadows **(A–D)** and alpine steppes **(E–H)**. AGB, BGB, SR, and H represent aboveground biomass, belowground biomass, species richness and Shannon-Wiener index, respectively.

**FIGURE 4:**
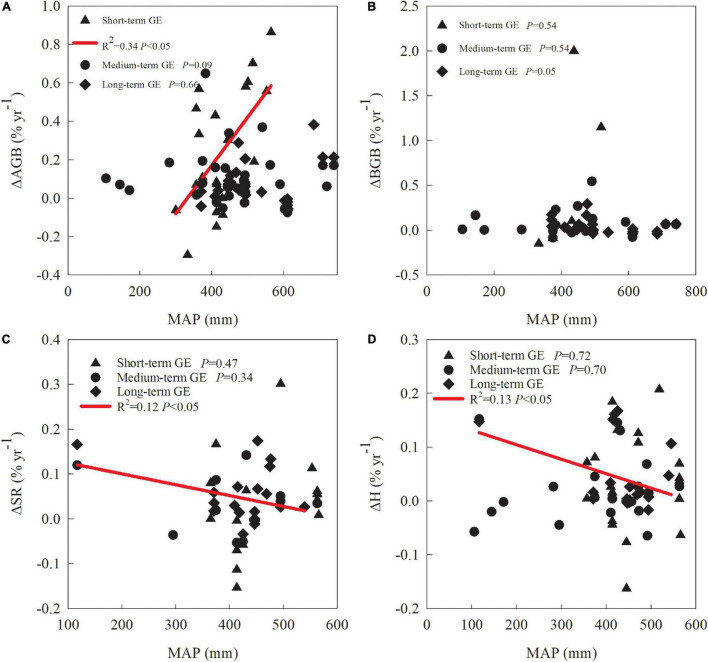
Relationships of the percentage of mean annual changes in above- and belowground biomass, species richness, and Shannon-Wiener index with mean annual precipitation under different grazing exclusion durations **(A–D)**. AGB, BGB, SR, and H represent aboveground biomass, belowground biomass, species richness and Shannon-Wiener index, respectively.

The percentage of mean annual changes in aboveground biomass (Figure 3) was significantly negatively related to GE duration in both alpine meadows (*R*^2^ = 0.06; *P* < 0.001; Figure 3A) and alpine steppes (*R*^2^ = 0.20; *P* < 0.001; Figure 3E). Additionally, there was also a significantly negative relationship between the percentage of mean annual changes in Shannon-Wiener index with GE duration in alpine meadows (*R*^2^ = 0.05; *P* < 0.001; Figure 3D), and a significantly negative relationship between the percentage of mean annual changes in species richness with GE duration in alpine steppes (*R*^2^ = 0.07; *P* < 0.01; Figure 3G). Interestingly, the percentage of mean annual changes in above- and belowground biomass both showed a significantly positive relationship with MAP for short-term GE (*P* < 0.05; Figures 4A,B). By contrast, the percentage of mean annual changes in both species richness and Shannon-Wiener index were significantly negatively associated with MAP for long-term GE (*P* < 0.05; Figures 4C,D).

## Discussion

### Changes in Plant Productivity and Diversity With Grazing Exclusion Duration

In this study, we found that short-term GE significantly increased plant aboveground biomass of alpine meadow, in consistent with previous studies (Shang et al., 2013; Liu et al., 2020b). It is mainly because that GE eliminates direct consumption of plant tissues by herbivore and thus facilitates a rapid restoration of vegetation after GE (Sun et al., 2018). The increase of plant biomass and litter material can enhance the input of organic matter into soil environment, reduce water loss caused by soil evaporation, and reduce soil erosion by improving soil surface roughness (Zhang et al., 2021). By contrast, aboveground biomass of alpine steppe exhibited no significant change with short-term GE, but an apparent increase with medium-term GE. The main reason might be that alpine meadow commonly experiences relatively higher precipitation than alpine steppe. Precipitation is tightly related to water availability for plant growth and has been proven a dominant factor driving vegetation restoration (Jia et al., 2015). Previous studies found that high precipitation greatly promoted grassland productivity (Liu et al., 2020a; Sun et al., 2020b). It explains that short-term GE significantly increases aboveground biomass of alpine meadow but has no significant effect on that of alpine steppe, which apparently recovers after medium GE. These findings agree with previous studies that patterns of vegetation recovery in response to GE are highly associated with precipitation (Wu et al., 2014a,^2017^). Precipitation may be a key factor in the restoration process of alpine steppes. This was supported by our results, as we found a significantly positive relationship between the efficiency of vegetation recovery with precipitation in this study. However, this positive relationship weakens as GE duration increases, and turns no significance in long-term GE. It implies that precipitation mainly promotes a rapid production restoration in early stage of GE, while the effect gradually loses when vegetation restores to some extent.

During short-term GE, plant belowground biomass shows no significant change in alpine meadow, while significantly increases in alpine steppe. It might be explained by the optimal partitioning hypothesis that plants maximize growth rate by allocating photosynthate among organs responding to habitat condition (Bloom et al., 1985; Sun et al., 2021b). The soils are commonly of relatively high soil nutrient availability in early stage of GE due to the fertilization effect of grazing through feces and urine (Bowman et al., 2003). Therefore, plants allocate more biomass to the aboveground part for a better access of light resources under abundant soil water and nutrient supply in alpine meadow, while they would partition more photosynthate to the belowground portion to maximize water uptake in relatively arid alpine steppe in early stage of GE (Dukes et al., 2005; Sun et al., 2021b). Additionally, the continuous increase in belowground biomass during long-term GE in this study indicates an intensifying competition for soil water and nutrient in the high-productivity grassland after long-term restoration.

We found increases in species richness with short-term GE in both alpine meadow and steppe, in consistent numerus previous studies (Tang et al., 2016; Guo et al., 2019; Liu et al., 2020b; Zhang et al., 2021). The logic is that GE eliminates trampling damage by herbivore and favors seedling establishment due to the high density of buried seeds (Ma et al., 2013), which leads to appearance of lost species induced by overgrazing, and annual species or short-lived perennials with higher colonization capacity rapidly establish during short-term GE (Zhao et al., 2019; Zhang et al., 2021). Plant interspecific facilitation plays a dominant role in species colonization during the early stage of GE (Bonet, 2004). However, an aggravating interspecific competition happens as a result of resource limitation in the high-productivity grassland after long-term GE, so that slow-growing and long-lived species with higher competitive advantages suppress or even exclude the less competitive species (Der Wal et al., 2004; Borer et al., 2014; Liu et al., 2019). Thus, there are remarked decreases in species richness and Shannon-Wiener index in long-term GE as reported in numerus previous studies (Oba et al., 2001; Wu et al., 2009; Kelemen et al., 2013).

### Practical Significance of Vegetation Restoration With Grazing Exclusion Duration

In this study, GE is well proved as an effective way for vegetation restoration indicated by the remarked improvement of productivity and species richness. However, the patterns of vegetation restoration with GE duration were diverse between alpine meadow and steppe. It suggests that the optimal GE duration should be different in different grassland types (Sun et al., 2020a). Given that vegetation of alpine meadow and steppe can restore to relatively high level with short- and medium-term GE, respectively, an appropriate use is advised after short-term GE in alpine meadow and medium GE in alpine steppe in order to avoid waste of grassland resource. Additionally, long-term GE reduces species richness since interspecific competition intensifies when productivity reaches environmental carrying capacity (Kenkel, 1988; Chesson, 2018). Moreover, we found that the restoring efficiency of vegetation restoration turns lower with GE duration increasing. According to these findings, we argue that GE should be ceased after a long period (> 8 years) because fences can be useful tools but only when they are transitional and impermanent (Sacha, 2020).

Plant diversity is very important for the development of animal husbandry, livelihood of herdsmen, and social production in the Tibetan Plateau. Curbing biodiversity loss is a cornerstone of Sustainable Development Goal 15 of the United Nations’ 2030 Agenda for Sustainable Development. In view of this, the maintenance of biodiversity through the removal of grazing exclusion should be encouraged. Transient grazing exclusion may make sense, but we need better understanding of their impact on biodiversity is needed.

In this study, restricted by experimental conditions, there was one plot for each of the treatments (grasslands with different years of GE), which may result in an excessively high pseudo-repetition rate. In addition, the effects of climate change were not considered, which may have played an important role in plant growth and community structure. These potential problems may limit assessment of the impact of grazing exclusion on grassland restoration in different years. Therefore, climate change and other factors should be considered on a long-term time scale in the future to continuously monitor the impact of grazing exclusion duration on restoration efficiency of degraded grassland.

## Conclusion

Our results showed that grazing exclusion was an effective way of vegetation restoration in alpine meadows and alpine steppes, however, the effect of grazing exclusion duration on vegetation restoration was different. Specifically, aboveground biomass is significantly increased with short- and medium-term grazing exclusion in alpine meadows, probably due to the relatively higher precipitation in alpine meadows improving the water availability for plant growth. In line with the optimal partitioning hypothesis, belowground biomass has no significant change with short-term grazing exclusion in alpine meadows, while significantly increases for a competition of water in the relatively arid alpine steppes. Plant species richness was significantly increased with medium- and long-term GE in alpine meadows, while was significantly increased with all duration of GE in alpine steppes. The Shannon-Wiener index are significantly increased with short-, medium- and long-term grazing exclusion in both alpine meadows and alpine steppes. It can be explained by the resource-supply trajectory regulating interspecific competition and a further invasion or arrival of plant species during long-term vegetation restoration with grazing exclusion. Given these findings and the fact that the efficiency of vegetation restoration by grazing exclusion gradually decreases with increasing grazing exclusion duration, medium-term grazing exclusion are suitable for vegetation restoration in alpine meadows and alpine steppes. In addition to vegetation restoration, soil that plays an important role in ecosystem functions and services cannot be ignored in grassland restoration. Therefore, further more comprehensive assessments of restoration efficiency including two aspects of both plant and soil with grazing exclusion duration are needed in alpine meadows and alpine steppes in future.

## Data Availability Statement

The raw data supporting the conclusions of this article will be made available by the authors, without undue reservation.

## Author Contributions

TZ and WZ conceived the study, wrote the manuscript, reviewed, and revised the manuscript. TZ and TH collected and analyzed the data. TZ and SF drew the graphs. All authors contributed to the article and approved the submitted version.

## Conflict of Interest

The authors declare that the research was conducted in the absence of any commercial or financial relationships that could be construed as a potential conflict of interest.

## Publisher’s Note

All claims expressed in this article are solely those of the authors and do not necessarily represent those of their affiliated organizations, or those of the publisher, the editors and the reviewers. Any product that may be evaluated in this article, or claim that may be made by its manufacturer, is not guaranteed or endorsed by the publisher.

## Footnotes

1
www.getdata-graph-digitizer.com/

2
http://www.geodata.cn
