# Supplementary material for: Plant Community Traits Respond to Grazing Exclusion Duration in Alpine Meadow and Alpine Steppe on the Tibetan Plateau
Source: Front Plant Sci. 2022 Jul 4;13:863246. doi: 10.3389/fpls.2022.863246 (PMC9291246; doi:10.3389/fpls.2022.863246)
Supplement: Supplementary file 1 [file Table_1.DOCX]

**Table S1** The variation of above-ground biomass (AGB), below-ground biomass (BGB), species richness (SR) and Shannon-wiener index (H) in alpine meadow and alpine steppe. The Mean, Min, and Max are the average, minimum, and maximum values, respectively.

| Type | Value | AGB (g/m^2^) | BGB(g/m2) | SR | H |
| --- | --- | --- | --- | --- | --- |
| Alpine meadow | Min | 2.84 | 11.16 | 2.01 | 0.38 |
|  | Mean | 199.95 | 1654.82 | 16.92 | 2.2 |
|  | Max | 1299.2 | 10274 | 42.08 | 10.14 |
| Appine steppe | Min | 8 | 20 | 0.83 | 0.38 |
|  | Mean | 122.92 | 222.54 | 7.76 | 1.56 |
|  | Max | 531.57 | 761.14 | 27 | 2.9 |
