# Supplementary material for: Plant Community Traits Respond to Grazing Exclusion Duration in Alpine Meadow and Alpine Steppe on the Tibetan Plateau
Source: Front Plant Sci. 2022 Jul 4;13:863246. doi: 10.3389/fpls.2022.863246 (PMC9291246; doi:10.3389/fpls.2022.863246)
Supplement: Supplementary file 2 [file Table_2.DOCX]

**Table S2** List of all the references used in the meta-analysis

[1] Wang S, Fan J, Li Y, et al. Effects of Grazing Exclusion on Biomass Growth and Species Diversity among Various Grassland Types of the Tibetan Plateau[J]. Sustainability, 2019, 11(6).

[2] Yao X, Wu J, Gong X, et al. Effects of long term fencing on biomass, coverage, density, biodiversity and nutritional values of vegetation community in an alpine meadow of the Qinghai-Tibet Plateau[J]. Ecological Engineering, 2019, 130:80-93.

[3] Dan Z, Bai M, Duo J, et al. Effect of livestock exclusion duration years on plant and soil properties in a Tibetan alpine meadow[J]. Pratacultural Science, 2018.

[4] Wang X, Song N, Yang X, et al. Grazing exclusion-induced shifts, the relative importance of environmental filtering, biotic interactions and dispersal limitation in shaping desert steppe communities, northern China[J]. Journal of Arid Land, 2018.

[5] Chen J, Luo Y, Xia J, et al. Divergent responses of ecosystem respiration components to livestock exclusion on the Qinghai Tibetan Plateau[J]. Land Degradation and Development, 2018.

[6] Zhao J, Sun F, Tian L. Altitudinal pattern of grazing exclusion effects on vegetation characteristics and soil properties in alpine grasslands on the central Tibetan Plateau[J]. Journal of Soils and Sediments, 2018:1-12.

[7] Wang L, Yang Y. The Influence of the Fenced Enclosure on the Vegetation Community Characteristics of Alpine Meadow Grassland in the Eastern Part of the Qinghai-Tibet Plateau[J]. Journal of Grassland and Forage Science, 2017.

[8] Li W, Cao W, Wang J, et al. Effects of grazing regime on vegetation structure, productivity, soil quality, carbon and nitrogen storage of alpine meadow on the Qinghai-Tibetan Plateau[J]. Ecological Engineering, 2017, 98(Complete):123-133.

[9] Xue H, Luo D, Wang H, et al. Effects of free grazing or enclosure on soil nematodes in alpine meadows in North Tibet, China. 2017.

[10] Ling L, He H, Wei Y, et al. Response of vegetation community structure, soil carbon sequestration, and water-holding capacity in returning farmland to grassland plots, in the agropastoral transitional zone in the Three Rivers Source Region[J]. Pratacultural Science, 2017.

[11] Wu J, Wang X D. Effect of Enclosure Ages on Community Characters and Biomass of the Degraded Alpine Steppe at the Northern Tibet[J]. Acta Agrestia Sinica, 2017.

[12] Zeng Q, An S, Liu Y. Soil bacterial community response to vegetation succession after fencing in the grassland of China[J]. Science of the Total Environment, 2017, 609(dec.31):2-10.

[13] Hong J, Ma X, Wang X. Leaf meristems: an easily ignored component of the response to human disturbance in alpine grasslands[J]. Ecology & Evolution, 2016:2325-2332.

[14] Yang Y, Li H, Zhang L, et al. Effects of Fencing Measurement on Vegetation Community Structure and Soil Water-holding Capacity in Batang Alpine Meadow[J]. Mountain Research, 2016.

[15] Liu Y, Zhang D, Zhang Y, et al. Evaluation of restoration effect in degraded alpine meadow under different regulation measures[J]. Transactions of the Chinese Society of Agricultural Engineering, 2016, 32(24):268-275.

[16] Liu X, Zhang X, Zhang L, et al. Effects of exclousure duration on the community structure and species diversity of an alpine meadow in the Qinghai-Tibet Plateau[J]. Acta Ecologica Sinica, 2016, 36(16).

[17] Ren Y, Zhou Y, Jing X, et al. Effect of Enclosure on Species Diversity and Productivity of Degraded Alpine Meadow in Tibet[J]. Guizhou Agricultural Sciences, 2015.

[18] Hong J T, Wu J, Wang X. Effects of grazing and fencing on Stipa purpurea community biomass allocation and carbon, nitrogen and phosphorus pools on the northern Tibet Plateau alpine[J]. Pratacultural Science, 2015.

[19] Zhang Y, Zhao W. Vegetation and soil property response of short-time fencing in temperate desert of the Hexi Corridor, northwestern China[J]. Catena, 2015.

[20] Lu X, Yan Y, Sun J, et al. Carbon, nitrogen, and phosphorus storage in alpine grassland ecosystems of Tibet: effects of grazing exclusion[J]. Ecology and Evolution, 2015, 5(19).

[21] Yan Y, Lu X. Is grazing exclusion effective in restoring vegetation in degraded alpine grasslands in Tibet, China? [J]. PeerJ, 2015, 3(6):e1020.

[22] Mao S, Wu Q, Zhu J, et al. Response of the maintain performance in alpine grassland to enclosure on the Northern Tibetan Plateau[J]. Acta Prataculturae Sinica,2015, 57(1):75-77.

[23] Feng-Xia L I, Xiao-Dong L I, Zhou B R, et al. Effects of grazing intensity on biomass and soil physical and chemical characteristics in alpine meadow in the source of three rivers[J]. Pratacultural Science, 2015.

[24] Zheng W, Dong Q, Li S, et al. Dynamics of plant community characteristics of alpine steppe under enclosure around Qinghai Lake[J]. Pratacultural Science, 2014.

[25] Zhao K, Yang X, Ma H, et al. Analyses on Community Characteristics and Soil Microorganism Dynamics during Ecological Restoration of *Sophora moorcroftiana* in the Semi-Arid Valley of Lhasa [J]. Scientia Silvae Sinicae, 2013, 49(002):15-20.

[26] Li Z, Ming Z, Li G, et al. Effects of Enclosure on Revegetation of Degradation Swamp Wetland in Hequ Stud-farm[J]. Chinese Agricultural Science Bulletin, 2013.

[27] Liu X, Wu Q, Li H, et al. A Comparison of the Vegetation/Soil Carbon Density and Net Ecosystem CO_2 Exchange of Alpine Meadow with Different Enclosure Durations[J]. Journal of Glaciology & Geocryology, 2013, 35(4):848-856.

[28] Zhang W, Gan Z, Li Y, et al. Effects of Banning Grazing and Delaying Grazing on Species Diversity and Biomass of Alpine Meadow in Northern Tibet[J]. Journal of Agricultural Science and Technology, 2013, 15(3):143-149.

[29] Ma M, Zhou X, Du G. Effects of disturbance intensity on seasonal dynamics of alpine meadow soil seed banks on the Tibetan Plateau[J]. Plant & Soil, 2013, 369(1-2):283-295.

[30] Fan Y, Hou X, Shi H, et al. Effects of grazing and fencing on carbon and nitrogen reserves in plants and soils of alpine meadow in the three headwater resource regions[J]. Russian Journal of Ecology, 2013, 44(1):80-88.

[31] Miao F, Guo Y, Miao P, et al. Influence of enclosure on community characteristics of alpine meadow in the northeastern edge region of the Qinghai-Tibetan Plateau[J]. Acta Prataculturae Sinica, 2012.

[32] Fan Y, Hou X, Shi H, et al. The response of carbon reserves of plants and soils to different grassland managements on alpine meadow of three headwater source regions[J]. Grassland & Turf, 2012.

[33] Wei D, Xu R, Wang Y, et al. Responses of CO2, CH4 and N2O fluxes to livestock exclosure in an alpine steppe on the Tibetan Plateau, China[J]. Plant & Soil, 2012, 359(1-2):45-55.

[34] Zhao J, Biao Q, Duo J, et al. Effects of short-term enclose on the community characteristics of three types of degraded alpine grasslands in the north Tibet[J]. Pratacultural Science, 2011.

[35] Gao Y, Zeng X, Schumann M, et al. Effectiveness of Exclosures on Restoration of Degraded Alpine Meadow in the Eastern Tibetan Plateau[J]. Arid Land Research and Management, 2011, 25(2):164-175.

[36] Yan S, Zhou Z, Qin Y, et al. Characteristics of nitrogen contents under different land use conditions in Alpine grassland of Maqu [J]. Acta Prataculturae Sinica, 2010, 19(002):153-159.

[37] Liu D, Ma Y, Zhang D, et al. Impact of enclosure on community characters of sowed Elymus nutans grassland in "black soil Land"[J]. Pratacultural Science, 2009.

[38] Wang W, Zeng Z, Yin H, et al. Effects of different land management measures on vegetation productivity on alpine *Kobresia* meadow[J]. Journal of Lanzhou University (Natural Sciences), 2009.

[39] Shang Z, Ma Y, Long R, et al. Effect of fencing, artificial seeding and abandonment on vegetation composition and dynamics of 'black soil land' in the headwaters of the Yangtze and the Yellow Rivers of the Qinghai‐Tibetan Plateau[J]. Land Degradation & Development, 2008, 19(5).

[40] Shi F, Chen H, Wu Y, et al. Effects of livestock exclusion on vegetation and soil properties under two topographic habitats in an alpine meadow on the eastern Qinghai-Tibetan Plateau[J]. Polish Journal of Ecology, 2010, 58(1), 125-133.

[41] Lu X, Yan Y, Sun J, et al. Short-term grazing exclusion has no impact on soil properties and nutrients of degraded alpine grassland in Tibet, China[J]. Solid Earth Discussions, 2015, 7(3):2413-2444.

[42] Sun J, Ma B, Lu X. Grazing enhances soil nutrient effects: Trade-offs between aboveground and belowground biomass in alpine grasslands of the Tibetan Plateau[J]. Land Degradation & Development, 2017.

[43] Sun J, Liu M, Fu B, et al. Reconsidering the efficiency of grazing exclusion using fences on the Tibetan Plateau[J]. Science Bulletin, 2020, 65(16): 1405-1414.
